# Viral Evasion of Natural Killer Cell Activation

**DOI:** 10.3390/v8040095

**Published:** 2016-04-12

**Authors:** Yi Ma, Xiaojuan Li, Ersheng Kuang

**Affiliations:** 1 Institute of Human Virology, Zhongshan School of Medicine, Sun Yat-Sen University, No. 74, Zhongshan 2nd Road, Guangzhou 510080, China; mayi3@mail2.sysu.edu.cn (Y.M.); lixjuan3@mail.sysu.edu.cn (X.L.); 2 Key Laboratory of Tropical Disease Control (Sun Yat-Sen University), Ministry of Education, Guangzhou 510080, China

**Keywords:** natural killer cells, viral evasion, activating receptor, ligands, herpesvirus

## Abstract

Natural killer (NK) cells play a key role in antiviral innate defenses because of their abilities to kill infected cells and secrete regulatory cytokines. Additionally, NK cells exhibit adaptive memory-like antigen-specific responses, which represent a novel antiviral NK cell defense mechanism. Viruses have evolved various strategies to evade the recognition and destruction by NK cells through the downregulation of the NK cell activating receptors. Here, we review the recent findings on viral evasion of NK cells via the impairment of NK cell-activating receptors and ligands, which provide new insights on the relationship between NK cells and viral actions during persistent viral infections.

## 1. Introduction

Natural killer (NK) cells serve as the first line of innate defense against viral infection, and they rapidly and directly kill infected cells in the absence of antigen presentation and recognition. In response to stimuli from diverse sources, including infections, cytokines, stresses and other immune cells, NK cells exert the following distinct functions: (i) secrete perforin and granzyme to directly kill target cells; (ii) release cytokines to regulate immune responses; and (iii) couple death-inducing receptors to target cells and induce apoptosis [1,2]. NK-deficient individuals are highly susceptible to a variety of viral infections, illustrating the key role of NK cells in the defense against viral infection [3]. However, viruses have evolved various strategies to evade the NK cell recognition and destruction during acute and persistent viral infections.

An array of activating or inhibitory receptors on the surface of NK cells recognize the ligands of target cells, and the relative expression of these receptors and the outcome of their signal cascades determines NK cell activation and cytotoxicity [4]. Numerous activating or inhibitory NK cell receptors have been identified in NK cells; the activating receptors recruit adaptors that contain the intracellular immunoreceptor tyrosine-based activating motif (ITAM), whereas the inhibitory receptors contain the immunoreceptor tyrosine-based inhibitory motifs (ITIM), consequently, they transduce activating or inhibitory signal cascades, respectively [5].

A cluster of inhibitory receptors specifically binds to major histocompatibility complex (MHC) class I molecules, such as the inhibitory Ly49s family members in mice, the killer-cell immunoglobulin-like receptors (KIR) in humans, and the heterodimeric CD94-NKG2A receptor in both species that recognizes non-classic MHC class I molecules. These molecules allow NK cells to be regulated by self-MHC recognition and restrain the NK cell hyperactivity [5]. Therefore, the NK cells preferentially kill the infected cells in which the surface expression of MHC molecules and the antigen presentation are inhibited by viruses [6].

Four types of activating NK receptors recognize the different ligands: CD16 enables NK cells to exert antibody-dependent cell cytotoxicity; natural killer group 2 member D (NKG2D) recognizes a family of stress-induced ligands; natural cytotoxicity receptors (NCRs) are able to recognize pathogen-derived or induced ligands and tumor ligands; and the other receptors, including 2B4 (CD244), NKG2C, DNAM1 (CD226) and NKp80, recognize self-molecules [4]. All receptors recognize a variety of ligands on the surface of target cells, and the major ligands include atypical major MHC class I, MHC class I-related chain A (MICA), MHC class I-related chain B (MICB), UL16 binding proteins 1–6 (ULBP1–ULBP6) and some viral proteins [5,7]. Upon the association between receptors and ligands, the receptors activate Syk (spleen tyrosine kinase) or ZAP70 (zeta-chain associated protein kinase 70 kDa) tyrosine kinases through the adapters DAP12, FcεRIγ (also known as FcRγ) or CD3ζ, or they activate phosphatidylinositol-3-kinase (PI3K) through the adaptor DAP10 [5]. During their development and maturation, NK cell receptors recognize self-ligands to obtain self-tolerance for normal and healthy cells through the processes of selection and education [8,9].

During viral infection, the balance of NK activating or inhibitory receptors shifts toward NK cell activation and increased cytotoxicity, whereas viruses employ complex mechanisms to reverse NK cell activation and maintain NK cell quiescence. Downregulation of MHC class I molecules by viruses prevents antigen presentation and reduces the immune response; however, it increases the susceptibility to NK cell recognition and destruction [6]. Viruses possess more effective and distinct strategies to escape from NK cell immunity, including stimulating the inhibitory receptors and disrupting the activating receptors.

Several viruses are able to inhibit NK cell activation through inhibitory receptors. Murine cytomegalovirus (MCMV) encoded MHC-I-like m157 in infected cell surfaces acts as a ligand of inhibitory Ly49C receptor, and their binding hampers NK cell activation. This outcome results in the evasion from NK cell clearance during MCMV infection in mice [10,11]. In humans, human leukocyte antigen (HLA)-C is capable of inhibiting NK cell cytotoxicity via inhibitory KIR receptors in human immunodeficiency virus type 1 (HIV-1) infection [12]. HLA-C presents HIV p24 epitopes to KIR receptors and engages KIRs on NK cells; therefore, it inhibits NK cell function [13]. Additionally, the epitopes of human cytomegalovirus (HCMV) glycoprotein UL40 are presented by HLA-E to NK cells via CD94/NKG2A receptor, by which protects the infected cells from NK cell killing [14]. The natural selection of variations provides a novel viral escape through inhibitory NK cell receptors [14,15]. However, knowledge on this strategy of viral evasion remains limited so far.

**Figure 1:**
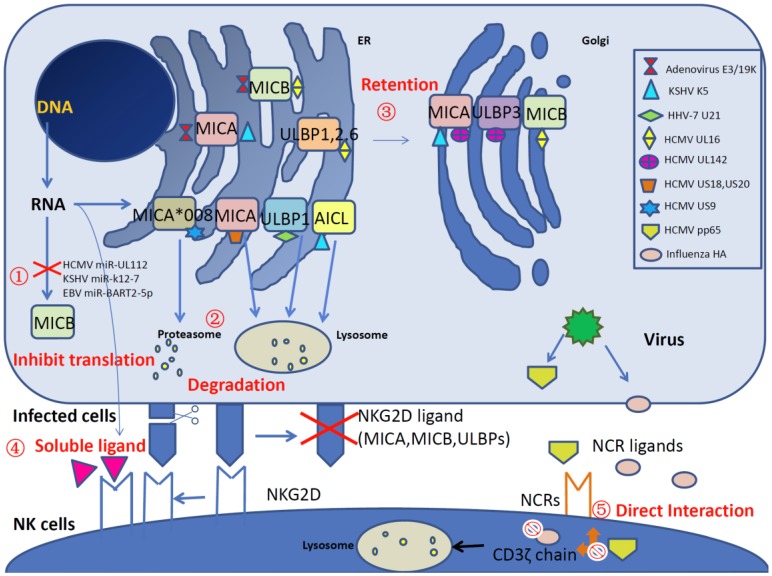
A schematic overview of viral interference with natural killer (NK) cell-activating receptors and ligands. A variety of viral products disrupt NK cell activation by impairing the natural killer group 2 member D (NKG2D) receptors and natural cytotoxicity receptors (NCRs) (NKp30, NKp44 and NKp46) and their ligands (major histocompatibility complex (MHC) class I-related chain A (MICA), MHC class I-related chain B (MICB) and UL16 binding proteins (ULBPs)) at different levels, as described below. ① miRNA-related ligand inhibition. Human cytomegalovirus (HCMV) miR-UL112, Kaposi’s sarcoma-associated herpesvirus (KSHV) miR-K12-7 and Epstein–Barr virus (EBV) miR-BART2-5p specifically inhibit MICB translation. ② Degradation of NKG2D ligands. KSHV K5 downregulates the cell surface expression of MICA and the NKp80 ligand activation-induced C-type lectin (AICL). HCMV proteins US18, US20 and HHV-7 protein U21 reduce the level of MICA by lysosomal degradation. Additionally, HCMV US9 targets MICA*008 for proteasomal degradation. ③ Retention of NKG2D ligands inside cells. Adenovirus E3/19K retains MICA and MICB in the endoplasmic reticulum (ER), HCMV UL142 downregulates MICA and ULBP3 by increasing retention in the *cis*-Golgi apparatus. Additionally, HCMV UL16 retains MICB, ULBP1, ULBP2 and ULBP6 in ER/*cis*-Golgi via direct interaction. ④ Competitive binding with NKG2D by soluble ligands. Orthopoxvirus MHC class I-like protein (OMCP) acts as an antagonist by binding to NKG2D and reduces NKG2D-mediated recognition. In addition, soluble NKG2D ligands are released by cellular matrix metalloproteinase in human immunodeficiency virus (HIV)-infected CD4+ T cells and impair NK cell recognition. ⑤ Inhibition of NCRs. The direct interaction between HCMV pp65 and NKp30 causes the dissociation between NKp30 and CD3ζ and then inhibits the activation of the signal cascade. In addition, influenza hemagglutinin (HA) induces the lysosomal degradation of CD3ζ and therefore inhibits NK cell cytotoxicity.

Emerging research has revealed many new strategies of viral evasion through effects on NK cell-activating receptors, including NKG2D receptors and NCRs (Figure 1). Here, we review the impairment of NK cell-activating receptors and ligands by viruses and further discuss the unique aspects of viral evasion of NK cell recognition and destruction, which provides novel insights on the struggles between NK cells and viruses during persistent viral infection.

## 2. NKG2D-Mediated Evasion

NKG2D is a type-II transmembrane-anchored C-type lectin-like receptor on all NK cells and on a majority of natural killer T (NKT) cells [16]. It is the most important activating receptor responsible for the viral infections, and its ligands are directly induced by viral infection due to the expression of viral products and the induction of interferons and cytokines. NK cells are activated by conjugating NKG2D to ligands, primarily MICA, MICB and ULBP1-6 in humans. These ligands are normally expressed on the surface of the majority of healthy cells at a level that is below the threshold of NK cell activation, however, they are upregulated by DNA damage, cell transformation and viral infection [16]. Upon the association between receptors and ligands, NKG2D complexes are coupled to PI3K or Grb2 through the DAP10 adaptor or recruited to Syk or ZAP70 tyrosine kinases by the DAP12 adaptor, leading to the NK cell activation and cytokine secretion.

Viruses have developed a series of strategies to prevent the presence of NKG2D or ligands on the cell surface and to evade NKG2D-mediated NK cell recognition and destruction, as described below and summarized in Table 1.

### 2.1. Viral Protein-Based Inhibition of NKG2D Ligands

A variety of viral proteins are capable of directly reducing the presence of NKG2D ligands on the cell surface through effects on their transportation, degradation inside the cells or distribution on the cell surface.

Several viral proteins are able to keep NKG2D ligands inside cells and prevent their surface expressions. HCMV glycoprotein UL16 selectively binds to ULBP1, ULBP2, ULBP6 and MICB but not to MICA, ULBP3, ULBP4 or ULBP5 and is able to increase their retention inside the ER/*cis*-Golgi compartment and consequently reduce their expression at the cell surface [18,19]. These UL16-binding ligands all share a similar binding motif and substitutions at this region prevent their binding to UL16 [46]. This common structure allows for a broad inhibition of the NKG2D ligands and counteracts their diversification. Alternatively, the surface expressions of MICA and ULBP3 are downregulated by HCMV UL142 through an increase in their retention in the *cis*-Golgi apparatus [20,21]. This strategy is also utilized by adenoviruses where the E3/19K protein sequesters MICA and MICB in the endoplasmic reticulum [27].

The degradation of NKG2D ligands is another effective strategy to inhibit NK cell activation. HCMV US18 and US20 target MICA for lysosomal degradation, reducing NK engagement with virally infected cells [23]. This type of evasion is employed by human herpesvirus-7 (HHV-7) where the U21 protein reroutes ULBP1 to the lysosomal compartment for degradation and downregulates the surface expression of MICA and MICB through destabilization [24]. In addition to lysosomal degradation, the HCMV US9 protein selectively downregulates the truncated MICA*008 allele, which is distinct from full-length alleles, via proteasomal degradation during HCMV infection [22]. Furthermore, the Kaposi’s sarcoma-associated herpesvirus (KSHV) ubiquitin E3 ligase K5 ubiquitinates MICA, MICB and the NKp80 ligand activation-induced C-type lectin (AICL), which induces their proteasomal degradation and reduces their surface expression, eventually enabling the virus to escape from destruction by NK cells [26].

NKG2D ligands are also able to be downregulated at the level of synthesis. MICA surface expression during vesicular stomatitis virus (VSV) infection is inhibited at the early post-transcriptional level because MICA mRNA expression is upregulated and its translation activity is not affected [36]. However, the viral products responsible for this inhibition remain unknown. Additionally, the HBV surface antigen induces the expression of several cellular microRNAs (miRNAs) to suppress MICA and MICB expression [28]. This is a novel mechanism in which cellular miRNAs are utilized by viruses to suppress NK cell activation.

Finally, some viral proteins suppress NKG2D ligands via undefined mechanisms. It has been shown that HIV infection reduces the expression of MICA, ULBP1 and ULBP2 via inhibition by Nef [29], downregulates the co-activating ligand NK, T and B cell antigen (NTB-A) via inhibition by Vpu [32,33] and downregulates the DNAM-1 ligand poliovirus receptor (PVR) via inhibition by both Nef and Vpu [30,31]. However, how these HIV proteins downmodulate these ligands has not been documented. Similarly, hepatitis C virus (HCV) NS2 and NS5B reduce MICA and MIC expression in infected hepatoma cells via an unknown mechanism [34]. Although the phosphorylation of C/EBP-β (CCAAT-enhancer-binding protein-β) is inhibited during HCV infection, it is not yet known whether this directly affects MICA and MIC expression. Additionally, different patterns of inhibition of NKG2D ligand expression are observed during infection by herpes simplex virus (HSV) and varicella-zoster virus (VZV), two α-herpesviruses [17]. However, it is still unknown whether these proteins are suppressed at the level of synthesis or are degraded after synthesis or what viral proteins are responsible for this inhibition.

### 2.2. Viral MiRNA-Based Inhibition of NKG2D Ligands

A number of miRNA are expressed by DNA viruses, and the function of the majority of these small regulatory RNA molecules remains largely unknown [47,48]. Recent studies have demonstrated the role of viral miRNA in the regulation of NKG2D ligands. HCMV-miR-UL112 specifically binds to MICB-3′ untranslated regions (3′UTR) and inhibits MICB translation, and non-miR-UL112 viral infection leads to an increase in MICB at the cell surface and easier recognition and destruction by NK cells [41]. This miRNA-mediated inhibition of an NKG2D ligand is conserved in herpesviruses even though viral miRNAs exhibit poor sequence homology. Epstein–Barr virus (EBV) miR-BART2-5p and KSHV miR-k12-7 inhibit MICB translation and, therefore, recognition and destruction by NK cells in a similar manner [42]. Because viral miRNAs are expressed during latent infection, miRNA-mediated evasion might be critical for viral latency. In addition to herpesviruses, polyomavirus John Cunningham virus (JCV) and BK virus (BKV) 3p* miRNA downregulates the expression of ULBP3 and reduces NK cell recognition [43]. However, it is still uncertain whether additional viral miRNAs impair other NK cell-activating receptors and ligands.

### 2.3. Soluble NKG2D Ligands

In addition to the inhibition of surface NKG2D ligands, soluble NKG2D ligands generated during certain viral infections can also act as distinct evasion mechanisms to avoid NK cell recognition by obstructing the interaction between NKG2D receptors and ligands. Zoonotic orthopoxviruses secrete a soluble antagonist of NKG2D, called orthopoxvirus MHC class I-like protein (OMCP), which competitively binds with NKG2D and inhibits destruction by NK cells [44]. The soluble NKG2D ligands have been observed in chronic HIV infection. Studies have revealed that the release of soluble NKG2D ligands, including soluble MICA, MICB, and ULBP2, is mediated by cellular matrix metalloproteinase in HIV-infected CD4+ T cells [45]. The increase of soluble NKG2D ligands in the plasma provokes NKG2D downregulation on NK and CD8+ T cells and, therefore, impairs the functions of NK cells in the recognition and destruction of infected cells.

### 2.4. Cytokine-Mediated Inhibition of NKG2D Expression

The secretion of cytokines is regulated by viral infection, and these cytokines are capable of suppressing the expression of NKG2D and other activating receptors in NK cells. HCMV infection in peripheral blood mononuclear cells prevents surface NKG2D expression via the secretion of type I interferon (IFN), interleukin-12 (IL-12) and IFNγ and selectively impairs NK-cell cytotoxicity via the NKG2D receptor and its ligands [37]. During persistent HBV infection, the expression of NKG2D- and 2B4-activating receptors and the intracellular adaptor proteins DAP10 and SAP, respectively, are significantly decreased. Consequently, NK cell-mediated cytotoxicity is impaired. During this process, transforming growth factor-β 1 (TGFβ1) expression is greatly increased and is responsible for these inhibitory effects [39]. Similarly, HCV NS5A stimulates IL-10 production and, subsequently, inhibits IL-12 and triggers TGFβ production [38]. As result, chronic HCV infection downregulates NKG2D expression on circulating NK cells and impairs NK cell function. All of these findings indicate that the cytokines are important in the evasion of NK cell recognition by infecting viruses, which suggests that neutralizing these cytokines might be a promising therapeutic strategy against persistent viral infections.

Additionally, secreted small molecules also play a key role in viral inhibition of NK cell activation. Prostaglandin E2 (PGE2) is responsible for the inhibition of NKG2D and NKp30 expression on NK cells during KSHV infection, by which KSHV disrupts NK cell function and activation in infected patients [40]. Chemical screening has identified several putative regulators of MICA [49], indicating that additional natural compounds might also exhibit these functions. These findings suggest that the active small molecules are another type of agent involved in the regulation of NK cell immune surveillance and evasion during viral infection.

## 3. NCR-Mediated Evasion

NCRs, including NKp30, NKp44 and NKp46, belong to the immunoglobulin superfamily and are important NK cell-activating receptors. NKp30 and NKp46 are constitutively expressed on all NK cells, while NKp44 is only expressed on activated NK cells. The binding of NCRs to their ligands leads to destruction by the NK cell. However, only a few NCR ligands have been identified as virus-derived or virus-induced products [50]. On the other hand, NCRs and ligands are also inhibited by viruses, as briefly outlined below and summarized in Table 2.

CD3ζ chain is important for the transduction of NKp46- and NKp30-mediated activating signaling. It has been shown that the HCMV tegument protein pp65 downregulates NK cell destructive functions through NKp30 [51]. The direct interaction between pp65 and NKp30 causes the dissociation of the NKp30 and CD3ζ chain. Normally, pp65 is neither expressed on the cell surface nor secreted outside cells. However, the cytolysis of HCMV infected cells results in the release of pp65 and therefore the inhibition of NK cells. This CD3ζ chain is also decreased by influenza viruses as a way to evade NK cell immune defenses. Hemagglutinin (HA) on the membrane of infected cells can mediate recognition and lysis by NK cells [59,60]. Thereafter, free HA proteins are released and internalized into NK cells, leading to a downregulation of the CD3ζ chain through lysosomal degradation [53]. These processes damage the NKp46 and NKp30 activating signal transduction pathway and inhibit NK cell cytotoxicity.

Meanwhile, NCRs are directly disrupted by viral ligands. The recognition of influenza HA by NKp44 and NKp46 is sialic acid-dependent, and viral neuraminidase (NA) protein impairs this recognition by removing sialic acid residues from NKp46, which then reduces NKp44- and NKp46-mediated recognition and destruction by NK cells [54,55]. In addition, poxvirus HA present on the surface of infected cells disrupts the net effect of NKp46 and NKp30 by blocking NKp30-triggered activation at the late stages of poxviral infections [52], by which poxviruses escape from NK cell immune responses. In addition, HCV-infected cells are able to downregulate the surface expression of NKp30 in NK cells, which allows HCV infected cells to evade NK immune surveillance and establish a chronic infection [35,58].

NCR ligands are also modulated by other viruses through distinct mechanisms. KSHV open reading frames (ORF)54-dUTP pyrophosphatase (dUTPase) downregulates NKp44 ligands by interfering with intracellular trafficking [56]. This downregulation is not associated with its dUTPase activity and homologous EBV dUTPase lacks this function, indicating this is an evasion mechanism unique to KSHV. Similarly, by inhibiting NKp44 ligand expression, HIV Nef allows HIV to avoid destruction by NK cells [57].

Information about this important viral strategy for evading NK cell activation is limited. Compared with the pathways for viral evasion of NKG2D receptors and ligands, few viral or cellular NCR ligands have been characterized, and this restricts advances in understanding the processes of the activation and evasion of NCR-mediated recognition and cytotoxicity by viruses.

## 4. Evasion of NK Cell Adaptive Responses

Memory is one of the features of the adaptive immune response of antigen-specific T and B lymphocytes, which provide the ability to evoke a rapid and effective response to secondary infections [61]. Memory is not generally thought to be presented in NK cells. However, emerging studies have shown that NK cells possess memory-like, antigen-specific, long-lived adaptive immune responses [62,63,64]. During MCMV infection, the NK cell-activating Ly49H receptor recognizes the MCMV m157 glycoprotein and then generates long-lived memory NK cells [65,66]. These memory Ly49H+ NK cells reside in the spleen and liver for several months and possess the ability to self-renew and recall antigen-specific responses. The generation of antigen-specific memory NK cells has also been observed in several other viral infections, such as influenza, VSV and HIV [67]. The memory-like NK cells have been confirmed in patients with CMV infection, but not in those with HSV-I/II infection. Likewise, the generation of an adaptive NK cell memory is associated with multiple protein deficiencies through epigenetic silence, including SYK and FcRγ deficiencies [68,69]. These studies indicate that NK cells possess a type of adaptive immunity. Although it remains uncertain whether viruses can escape from antigen-specific NK cell adaptive immunity, studies have shown that MCMV m157 viruses rapidly mutate and fail to activate Ly49H-positive NK cells after experiencing selective pressure from Ly49H+ NK cells [70,71], which suggests that MCMV might be able to escape from the NK cell adaptive immune response via antigen variation. A recent finding reveals that an enhanced autophagy is essential for the generation of NK cell memory during MCMV infection [72,73]. Many viral products are capable of modulating autophagy through diverse mechanisms [74,75]. It would be interesting to determine whether the NK cell adaptive memory is suppressed by viral infections and cytokines through autophagy. However, there is not yet sufficient evidence to hypothesize whether viruses in general are capable of evading NK cell adaptive immune responses.

## 5. Evasion of NK Cell Activation by γ-Herpesvirus

Both KSHV and EBV are highly homologous oncogenic γ-herpesviruses that have the ability to establish a long-term latent infection in humans [76]. Their infections cause lymphoproliferative diseases that display latency in lymphoid tissues. Current research has shown that both share common strategies for immune evasion and signal transduction [77,78], and presumably, NK cell-activating receptors might be manipulated by both viruses via certain conserved strategies.

It has been shown that KSHV K5 is capable of preventing the surface expression of the ligands of both the NKG2D and NKp80 activation pathways [26], and KSHV ORF54 downregulates the ligand of the NK activating receptor NKp44 [56]. EBV LMP2A reduces MICA and ULBP4 in lymphoblastoid cell lines and inhibits recognition by CD8+ T cells [25], and this allows it to block its recognition and destruction by NK cells. Additionally, both EBV miR-BART2-5p and KSHV miR-k12-7 have been implicated in the downregulation of MICB expression [42], which represents another common strategy for avoiding NK cell activation. Compared with knowledge about the multiple strategies of HCMV to avoid recognition and destruction by NK cells, the understanding of the interference with receptors and ligands exhibited by both KSHV and EBV infections remains largely limited.

The reactivation from a latent infection sensitizes EBV-infected B cells to destruction by NK cells during the early stages and viral products counteract it at later stages [79,80]. Therefore, the different NK cell responses occur during the different stages of the viral lifecycle and the relationship between NK cell activation and viral evasion may influence the outcome of viral infection and diseases. Consequently, the selective modulation of NK cell receptors and ligands by viral products is of great importance in persistent infections and pathogenesis. Further systematic investigations, including gain-of-function screening using viral ORFs and miRNA libraries and corresponding loss-of-function studies, into NK cell responses and viral evasion strategies could provide novel insights toward both persistent viral infections and diseases.

In addition to KSHV and EBV, there are several other γ-herpesviruses, such as murine gammaherpesvirus 68, rhesus monkey rhadinovirus and herpesvirus saimiri. All of them infect animals and share a high genetic similarity, as well as a similar biological activity to that of human γ-herpesviruses. These viruses and animals provide economical and useful models to study NK cell immune responses and viral evasion during infection with γ-herpesviruses.

## 6. Future Perspective

NK cells not only directly recognize and kill viral-infected cells through their receptors but also provide an antigen-specific adaptive response to viral infections, which represents the first line of defense and a rapid immune response against viral infections. NKG2D and NCRs are well-characterized NK cell-activating receptors, and their receptor-ligand interactions leads to NK cell activation and cytokine production. In turn, viruses counterattack the recognition and destruction of NK cells by inhibiting NK cell-activating receptors and ligands, as described in the current review. As there are still considerable questions about these processes, further investigations are required to understand the relationship between viral infection and NK cell immune responses.

Due to the species-specificity of human NK cells as well as technical and ethical considerations, it is difficult to study NK cell antiviral immune responses and viral escape in humans. Fortunately, genetically humanized mice provide effective tools to study human immune responses and diseases [81,82]. Numerous humanized mice have been used in studies of HCV, HIV and EBV infections [83,84,85]. It is likely that these human NK cell-harboring models will be widely used in this field of study.

Furthermore, NK cells provide a promising venue for the development of cell-based therapeutic strategies for treating persistent viral infection and diseases. Disruptions in the viral evasion of NK cell immune responses increases NK cell cytotoxicity to viral infection [22,51], and a viral genome with a substitution to produce an NKG2D ligand can act as an attenuated viral strain and induce protective responses against viral challenges [86], which provides a promising approach to restrict persistent infections using the properties of vaccines. Further genetic manipulation in viral genomes and investigations into NK cell responses might provide additional clues for the application of such vaccines. Additionally, to utilize NK cells for therapeutic applications, there are still large numbers of questions to answer about the mechanisms of viral evasion and achieve clinical efficacy.

## 7. Open Questions

(1) Different viruses employ distinct strategies for immune evasion, and it remains to be determined whether there are additional mechanisms in other viruses to evade NK cell activation and avoid destruction. Additionally, the molecular details of the innate and adaptive functions of NK cells remain limited. Further investigation may reveal additional novel strategies for viral evasion of NK cell immunity.
(2) Although a large number of studies have confirmed viral evasion of adaptive and innate immune responses, the mechanisms of the viral evasion of the NK cell adaptive immune response still largely remain unknown. More studies are required to reveal whether viruses can avoid NK cell memory-like adaptive functions and, if so, how this process works.
(3) It is worth investigating additional viral effects on NKG2D and ligands in NKT- and T cell-based immune responses. NKG2D is not only expressed on NK cells but also on NKT cells and T cells, and modulation of NKG2D and ligands may contribute broadly to viral evasion.

## Figures and Tables

**Table 1:** Viral gene products and NKG2D-mediated evasion of NK cell defenses.

Virus	Viral Product	Mechanisms	References
**Viral proteins**			
HSV	?	Decreases MICA, ULBP2, ULBP3 and ULBP1 on the cell surface	[17]
VZV	?	Reduces ULBP2 and ULBP3 on the cell surface	[17]
HCMV	UL16	Retains ULBP1, ULBP2, ULBP6 and MICB in the ER/*cis*-Golgi	[7,18,19]
	UL142	Retains ULBP3 and MICA in the *cis*-Golgi apparatus	[20,21]
	US9	Induces MICA*008 proteasomal degradation	[22]
	US18, US20	Induces MICA lysosomal degradation	[23]
HHV-7	U21	Redirects ULBP1 to lysosomal degradation	[24]
		Downregulates expression of MICA and MICB	
EBV	LMP2A	Reduces the expression of MICA and ULBP4	[25]
KSHV	K5	Redistributes MICA to an intracellular compartment	[26]
		Induces AICL endolysosomal degradation	[26]
Adenovirus	E3/19K	Retains MICA and MICB in the ER	[27]
HBV	HBsAg	Downregulates MICA and MICB by inducing human miRNAs	[28]
HIV	Nef	Downregulates the cell surface abundance of MICA, ULBP1 and ULBP2	[29]
	Vpu, Nef	Downregulates the expression of NTB-A and PVR	[30,31,32,33]
HCV	NS2, NS5B	Downregulates MICA and MICB expression	[34]
	?	Downregulates NKG2D expression via cell-to-cell interaction	[35]
VSV	?	Suppresses MICA, MICB and ULBP2 expression	[36]
**Cytokines and secretory molecules**			
HCMV	?	Inhibits NKG2D/DAP10 expression through type I IFN and IL-12	[37]
HCV	NS5A	Downregulates NKG2D expression through inducing IL-10-TGFβ	[38]
HBV	?	Reduces NKG2D/DAP10 and 2B4/SAP expression through TGFβ	[39]
KSHV	?	Downregulates NKG2D expression through PGE2	[40]
**Viral miRNA**			
HCMV	miR-UL112	Inhibits MICB mRNA translation	[41]
EBV	miR-BART2-5p	Inhibits MICB mRNA translation	[42]
KSHV	miR-k12-7	Inhibits MICB mRNA translation	[42]
JCV, BKV	3p* miRNA	Inhibits ULBP3 mRNA translation	[43]
**Soluble receptor and ligands**			
Zoonotic orthopoxviruses	OMCP	Secretes soluble NKG2D ligand	[44]
HIV	?	Releases soluble NKG2D ligands via proteolytic shedding	[45]

HSV: herpes simplex virus; MICA: MHC class I polypeptide-related chain A; MICB: MHC class I polypeptide-related chain B; ULBP: UL16 binding protein; VZV: varicella-zoster virus; HCMV: human cytomegalovirus; ER: endoplasmic reticulum; HHV-7: human herpesvirus 7; EBV: Epstein–Barr virus; KSHV: Kaposi's sarcoma-associated herpesvirus; AICL: activation-induced C-type lectin; HBV: hepatitis B virus; miRNA: micro RNA; HIV: human immunodeficiency virus; HCV: hepatitis C virus; VSV: vesicular stomatitis virus; IFN: interferon; IL: interleukin; TGFβ: transforming growth factor beta; JCV: John Cunningham virus; BKV: BK virus; OMPC: orthopoxvirus MHC class I-like protein

**Table 2:** Viral gene products and NCR-mediated evasion of NK cell defenses.

Virus	Viral Product	Mechanisms	References
HCMV	pp65	Inhibits the dissociation of NKp30 and CD3ζ chain	[51]
Poxvirus	HA	Inhibits NKp30-triggered activation	[52]
Influenza Virus	HA	Inhibits NKp46 through lysosomal degradation of CD3ζ chains	[53]
	NA	Inhibits NKp44 and NKp46 recognition via the removal of sialic acid residues	[54,55]
KSHV	ORF54/dUTPase	Inhibits the NKp44 ligand by interfering with intracellular trafficking	[56]
HIV	Nef	Inhibits the NKp44 ligand through intracellular retention	[57]
HCV	?	Downregulates NKp30 expression in NK cells	[35,58]
